# GFF3sort: a novel tool to sort GFF3 files for tabix indexing

**DOI:** 10.1186/s12859-017-1930-3

**Published:** 2017-11-14

**Authors:** Tao Zhu, Chengzhen Liang, Zhigang Meng, Sandui Guo, Rui Zhang

**Affiliations:** Biotechnology Research Institute, Chinese Academy of Agricultural Sciences, Beijing, 100081 China

**Keywords:** GFF3, JBrowse, Visualization, Tabix

## Abstract

**Background:**

The traditional method of visualizing gene annotation data in JBrowse is converting GFF3 files to JSON format, which is time-consuming. The latest version of JBrowse supports rendering sorted GFF3 files indexed by tabix, a novel strategy that is more convenient than the original conversion process. However, current tools available for GFF3 file sorting have some limitations and their sorting results would lead to erroneous rendering in JBrowse.

**Results:**

We developed GFF3sort, a script to sort GFF3 files for tabix indexing. Specifically designed for JBrowse rendering, GFF3sort can properly deal with the order of features that have the same chromosome and start position, either by remembering their original orders or by conducting parent-child topology sorting. Based on our test datasets from seven species, GFF3sort produced accurate sorting results with acceptable efficiency compared with currently available tools.

**Conclusions:**

GFF3sort is a novel tool to sort GFF3 files for tabix indexing. We anticipate that GFF3sort will be useful to help with genome annotation data processing and visualization.

**Electronic supplementary material:**

The online version of this article (10.1186/s12859-017-1930-3) contains supplementary material, which is available to authorized users.

## Background

As a powerful genome browser based on HTML5 and JavaScript, JBrowse has been widely used since released in 2009 [1, 2]. According to its configuration document [3], it works by first converting genome annotation data in GFF3 file formats to JSON files by a built-in script “flatfile-to-json.pl”, and then rendering visualized element models such as genes, transcripts, repeat elements, etc. The main problem, however, is that this step is extremely time-consuming. The time is proportional to the number of feature elements in GFF3 files (Additional file 1). Even for small genomes like yeast (*Saccharomyces cerevisiae*), it takes ~10 s to finish the conversion. For large and deeply annotated genomes such as that of humans, the time increases to more than 15 min. In addition, through the conversion process, a single GFF3 file is converted to thousands of piecemeal JSON files, thus putting a heavy burden on the ability to back up and store data.

**Fig. 1:**
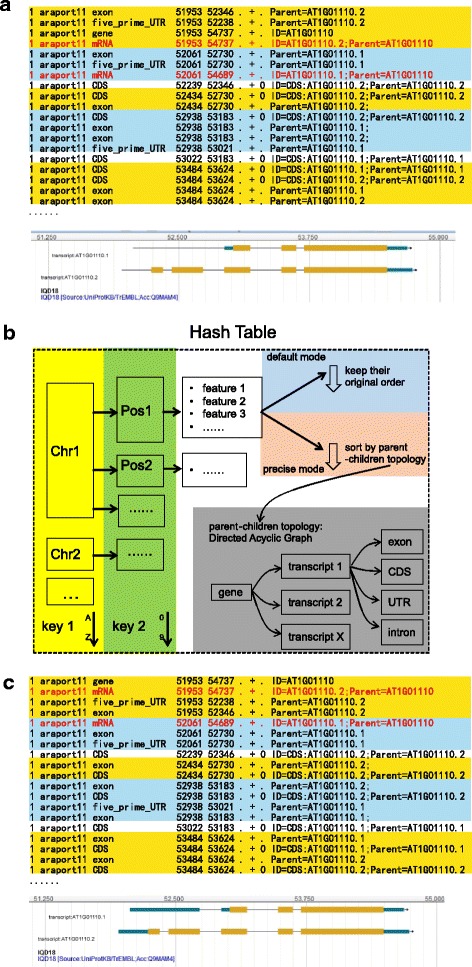
The motivation for, outlines of, and action effects of GFF3sort. **a** An example of incorrectly sorted GFF3 data and how this is rendered in JBrowse. Blocks with the same start position are marked in blue-yellow stripes. The two lines (mRNA) marked in red were placed after their sub-features (exon or UTR). Such an ordering leads to losing the first exon in JBrowse rendering results. See Additional file 2 for the full annotation lines. **b** Overview of GFF3sort. **c** An example of features sorted by GFF3sort and this is correctly rendered by JBrowse. In this example, the two lines (mRNA) marked in red are now placed before their sub-features, allowing JBrowse to render them correctly

In the recently released JBrowse version (v1.12.3), support for indexed GFF3 files has been added [4]. In this strategy, the GFF3 file is compressed with bgzip and indexed with tabix [5], which generates only two data files: a compressed file (.gz) and an index file (.tbi or.csi). Compared with the traditional processing protocol, the whole compression and index process could be finished within a few seconds even for large datasets such as the human genome annotation data (Additional file 1). The tabix tool requires GFF3 files to be sorted by chromosomes and start positions, which could be performed in the GNU sort program or the GenomeTools [6] package (see [7]). When dealing with feature lines in the same chromosome and start position, both of these tools may break ties or return a sort order where child features are placed ahead of their parent feature (Fig. 1a). Although this is still valid for tabix indexing, it would causing erroneous rendering in JBrowse [8] (Fig. 1a). Currently there is no additional options or arguments for current tools to break such tied features by parent-child relationship. In the absence of a suitable bug fix to JBrowse, an alternative sorting tool is needed to resolve this problem.

Here, we present GFF3sort, a novel tool to sort GFF3 files for tabix indexing. Compared with GNU sort and GenomeTools, GFF3sort produces sorting results that can be correctly rendered by JBrowse while still has comparable time and memory requirements. We anticipate that GFF3sort will be a useful tool to help with processing and visualizing genome annotation data.

## Implementation

GFF3sort is a script written in Perl. It uses a hash table to store the input GFF3 annotation data (Fig. 1b). For each feature, the chromosome ID and the start position are stored in the primary and secondary key, respectively. Features with the same chromosome and start position are grouped in an array in the same order of their appearance in the original GFF3 data. After sorting the hash table by chromosome IDs and start positions, GFF3sort implemented two modes to sort features within the array: the default mode and the precise mode (Fig. 1b). In most situations, the original GFF3 annotations produced by genome annotation projects have already placed parent features before their children. Therefore, GFF3sort returns the feature lines in their original order, which is the default behavior. In some situations where orders in the input file has not yet placed parent features before child features, GFF3sort would re-place them according to the parent-child topology using the sorting algorithm of directed acyclic graph [9], which is the most precise behavior but costs a little more computational time.

In order to test the performance of GFF3sort, the GFF3 annotation files of seven species, *Saccharomyces cerevisiae* (R64–1-1), *Aspergillus nidulans* (ASM1142v1), *Chlamydomonas reinhardtii* (INSDC v3.1), *Drosophila melanogaster* (BDGP6), *Arabidopsis thaliana* (Araport11), *Rattus norvegicus* (Rnor_6.0), and *Homo sapiens* (GRCh38), were downloaded from the ENSEMBL database [10]. All the tests were conducted on a SuperMicro® server equipped with 80 Intel® Xeon® CPUs (2.40GHz), 128 GB RAM, and running the CentOS 6.9 system. By default, CentOS 6.9 carries GNU sort v8.4, a relatively old version released in 2010. Therefore, we downloaded and installed a new version (v8.28) from the official repository of GNU Coreutils [11]. Both the old and the new version of GNU sort are used in benchmarking.

## Results and discussion

GFF3sort takes a GFF3 file as its input data and returns a sorted GFF3 file as output. Several optional parameters are provided such as turning on the precise mode, sorting chromosomes in different ways and properly dealing with inline FASTA sequences. Features sorted by GFF3sort are correctly rendered by JBrowse (Fig. 1c and Additional file 2).

In addition to providing a sort order that correctly renders in JBrowse, GFF3sort has also other advantages over traditional tools. Compared with the GNU sort program, GFF3sort can properly deal with GFF3-specific lines or directives that are preceded by the ‘##’ symbol, such as the topmost GFF version line, the sequence-region lines, and the embedded FASTA sequences. Compared with the GenomeTools, GFF3sort runs significantly faster (Additional file 1). In the default mode, GFF3sort saves ~70% running time in our seven test datasets. The precise mode takes longer to run but is still faster than GenomeTools, especially for large annotation data such as human. While keeping a high running speed, the memory consumption is still acceptable (Additional file 1). For the largest annotation dataset (the GRCh38 annotation version of human) with a ~400 MB GFF3 file, the memory usage of GFF3sort is ~758 MB, ~40% less than GenomeTools.

## Conclusions

In conclusion, GFF3sort is a novel tool to sort GFF3 files for tabix indexing and therefore can be used to visualize annotation data in JBrowse appropriately. It has a fast running speed compared with similar, existing tools. We anticipate that GFF3sort will be a useful tool to simplify data processing and visualization.

## Availability and requirements

Project name: GFF3sort.

Project home page: https://github.com/billzt/gff3sort

Operating system(s): Linux.

Programming language: Perl.

Other requirements: No.

License: No restrictions for academic users.

Any restrictions to use by non-academics: license needed.

## Additional files

Additional file 1: Benchmark data. This file displays: 1) the detailed running time of GFF3-to-JSON conversion and the bgzip-tabix process on our test datasets; 2) the detailed running time and 3) memory usage of GFF3sort, GNU sort (v8.4 and v8.28), and GenomeTools on our test datasets. (PDF 720 kb)

Additional file 2: The full GFF3 annotation lines used in Fig. 1a and c. It is the gene AT1G01110 extracted from the *Arabidopsis thaliana* (Araport11) annotation files. It includes three plain-text files: raw.gff3, GNUsort.gff3 (Fig. 1a), and GFF3sort.gff3 (Fig. 1c). (ZIP 2 kb)

## Abbreviations

GFF3: General Feature Format, version 3
HTML5: HyperText Markup Language, version 5
JBrowse: JavaScript-based genome browser
JSON: JavaScript Object Notation
