# Supplementary material for: GFF3sort: a novel tool to sort GFF3 files for tabix indexing
Source: BMC Bioinformatics. 2017 Nov 14;18:482. doi: 10.1186/s12859-017-1930-3 (PMC5686826; doi:10.1186/s12859-017-1930-3)
Supplement: Supplementary file 1 — Benchmark data. This file displays: 1) the detailed running time of GFF3-to-JSON conversion and the bgzip-tabix process on our test datasets; 2) the detailed running time and 3) memory usage of GFF3sort, GNU sort (v8.4 and v8.28), and GenomeTools on our test datasets. (PDF 720 kb) [file 12859_2017_1930_MOESM1_ESM.pdf]

Additional file 1 Benchmark data

Table S1. Comparison of the running time[1] of GFF3-to-JSON conversion and bgzip-tabix process.

**Data Source**	**Feature Number[2]**	**Running time (in seconds)**		
		**JSON conversion**	**bgzip + tabix**	**bgzip (8 threads) + tabix**
Saccharomyces cerevisiae (R64-1-1)	28,872	12.8	0.3	0.2
Aspergillus nidulans (ASM1142v1)	98,817	35.6	0.5	0.3
Chlamydomonas reinhardtii (INSDC v3.1)	291,092	99.2	1.2	0.7
Drosophila melanogaster (BDGP6)	479,987	157.4	1.8	0.9
Arabidopsis thaliana (Araport11)	791,564	267.5	3.1	1.4
Rattus norvegicus (Rnor_6.0)	1,023,357	411.5	4.5	2.2
Homo sapiens (GRCh38)	2,625,173	1,009.2	11.4	4.8

[1]The running time is measured by the GNU `time` tool. The commands are listed as follows.

- JSON conversion (JBrowse 1.12.3): `JBrowse/bin/flatfile-to-json.pl --gff input.gff3 --out out.dir --trackLabel label`
- bgzip + Tabix (both v1.3): `bgzip input.gff3 && tabix -p gff input.gff3.gz`
- bgzip (8 threads) + Tabix (both v1.3): `bgzip -@ 8 input.gff3 && tabix -p gff input.gff3.gz`

[2]Feature numbers are calculated by counting the line number of GFF3 files (excluding the comment lines started with a `#` symbol).

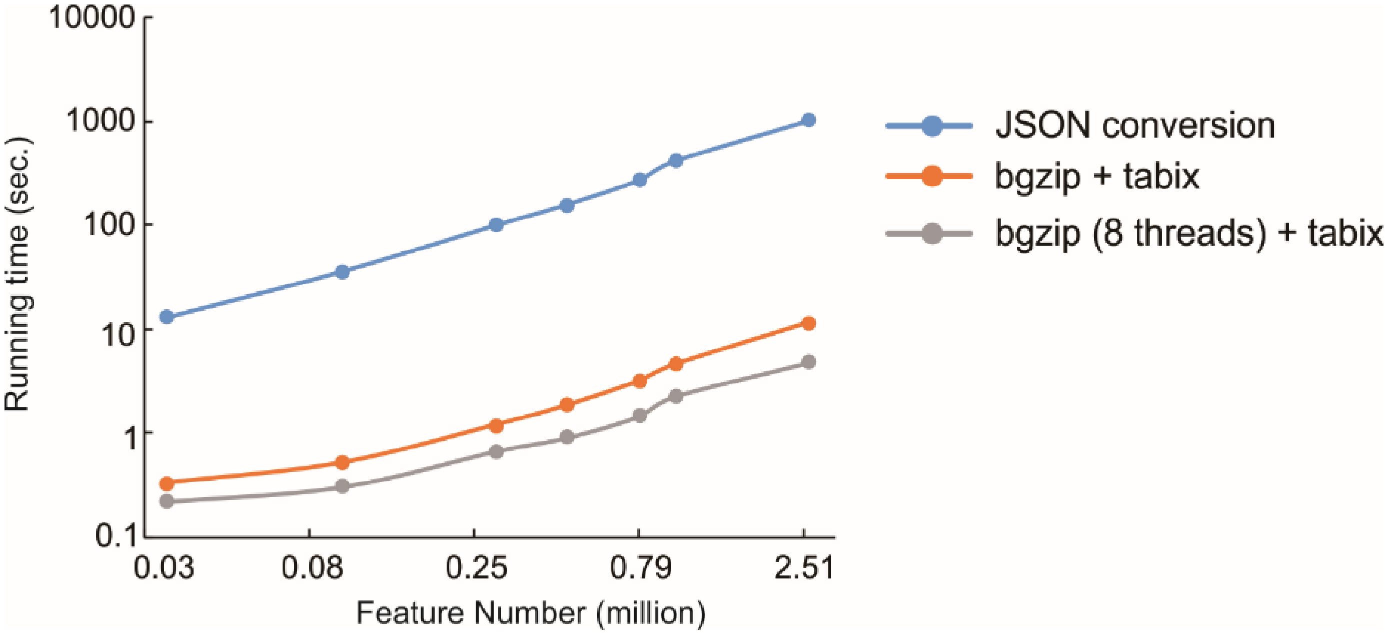

Figure S1. Comparison of the running time of GFF3-to-JSON conversion and bgzip-tabix process. The X and Y axes are transformed by LOG10.

Table S2. Comparison of the running time[1] of GFF3 sorting tools.

Data Source	Feature	Running time (in seconds)					
	Number	GNU sort (v8.4)	GNU sort (v8.28), 1 CPU	GNU sort (v8.28), 8 CPUs	GenomeTools	GFF3sort default mode	GFF3sort precise mode
Saccharomyces cerevisiae (R64-1-1)	28,872	0.6	0.07	0.07	0.6	0.2	0.6
Aspergillus nidulans (ASM1142v1)	98,817	2.6	0.22	0.21	1.8	0.6	1.8
Chlamydomonas reinhardtii (INSDC v3.1)	291,092	7.6	0.79	0.41	4.7	1.8	5.4
Drosophila melanogaster (BDGP6)	479,987	18.6	2	0.74	7.7	2.5	7.3
Arabidopsis thaliana (Araport11)	791,564	30.7	2.87	0.85	15.1	4.3	12.6
Rattus norvegicus (Rnor_6.0)	1,023,357	39.3	3.06	0.92	20.1	7.1	14.1
Homo sapiens (GRCh38)	2,625,173	113.7	10.76	2.71	67.9	15.6	40.2

[1]The running time is measured by the GNU `time` tool. The commands are listed as follows.

- GNU sort
  - (GNU coreutils v8.4): `sort -k 1,1 -k 4,4n input.gff3 >sort.gff3`
  - (GNU coreutils v8.28, 1 CPU): `sort -k 1,1 -k 4,4n --parallel=1 input.gff3 >sort.gff3`
  - (GNU coreutils v8.28, 8 CPUs): `sort -k 1,1 -k 4,4n --parallel=8 input.gff3 >sort.gff3`
- GenomeTools (v1.5.8): `gt gff3 -sortlines -retainids [input.gff3] >[sort.gff3]`

- GFF3sort (default mode): `gff3sort.pl [input.gff3] >[sort.gff3]`
- GFF3sort (precise mode): `gff3sort.pl --precise [input.gff3] >[sort.gff3]`

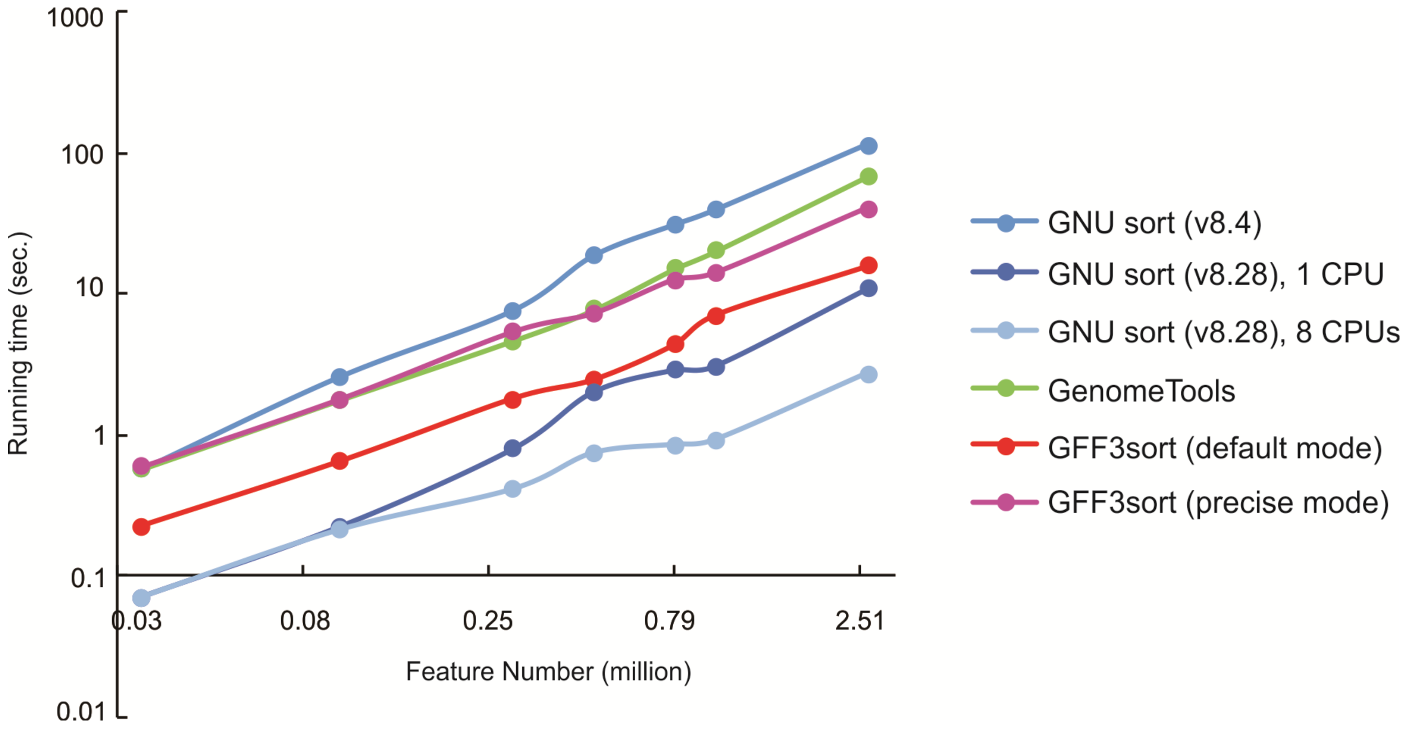

Figure S2. Comparison of the running time of GFF3 sorting tools. The X and Y axes are transformed by LOG10. GNU sort (v8.4), GenomeTools and GFF3sort cannot be parallelized and only use one CPU.

Table S3. Comparison of the memory usage[1] of GFF3 sorting tools.

Data Source	Size of the GFF3 file (MB)	Memory Usage (MB)				
		GNU sort (v8.4)	GNU sort (v8.28), 1 CPU	GNU sort (v8.28), 8 CPUs	GenomeTools	GFF3sort
Saccharomyces cerevisiae (R64-1-1)	5.2	9.7	9.4	8.8	20.4	13.5
Aspergillus nidulans (ASM1142v1)	15	21.4	23.4	21.4	52.2	36.1
Chlamydomonas reinhardtii (INSDC v3.1)	38	54.7	54.7	68.3	129.4	94.8
Drosophila melanogaster (BDGP6)	62	88.2	88.2	113.0	213.3	128.0
Arabidopsis thaliana (Araport11)	106	146.5	148.5	212.7	359.2	220.7
Rattus norvegicus (Rnor_6.0)	141	193.8	193.7	278.7	460.7	352.2
Homo sapiens (GRCh38)	404	539.7	539.7	748.3	1,260.8	757.7

[1]The memory usage is measured by the GNU `time` tool (by the `-v` option). The commands are listed as follows.

- GNU sort
  - (GNU coreutils v8.4): `sort -k 1,1 -k 4,4n input.gff3 >sort.gff3`
  - (GNU coreutils v8.28, 1 CPU): `sort -k 1,1 -k 4,4n --parallel=1 input.gff3 >sort.gff3`
  - (GNU coreutils v8.28, 8 CPUs): `sort -k 1,1 -k 4,4n --parallel=8 input.gff3 >sort.gff3`
- GenomeTools (v1.5.8): `gt gff3 -sortlines -retainids [input.gff3] >[sort.gff3]`

- GFF3sort (default mode): `gff3sort.pl [input.gff3] >[sort.gff3]`
- GFF3sort (precise mode): `gff3sort.pl --precise [input.gff3] >[sort.gff3]`

According to our test, the memory usage of GFF3sort in the default mode and the precise mode are almost the same (data not shown).

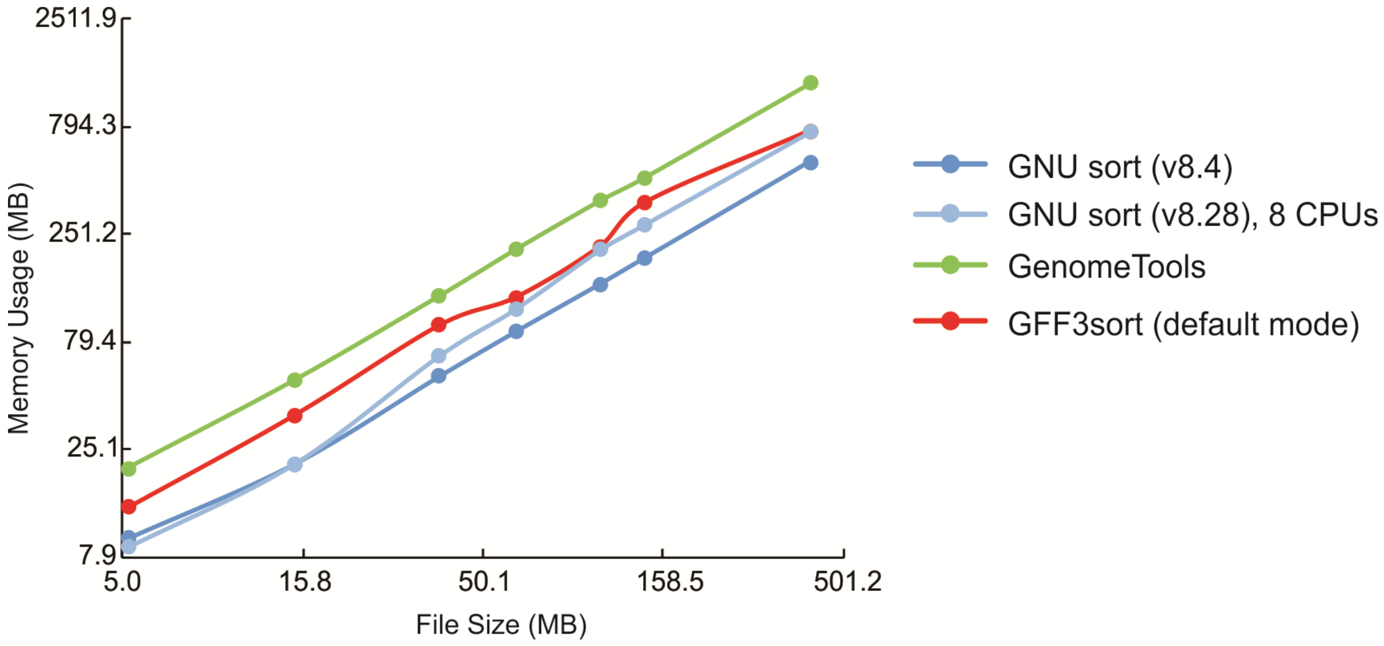

Figure S3. Comparison of the memory usage[1] of GFF3 sorting tools. The X and Y axes are transformed by LOG10. As the memory usage of GNU sort v8.4 and v8.28 (1 CPU) are almost the same (see Table S3), the curve for GNU sort v8.28 (1 CPU) is not shown.
